# A Circularly Polarized Broadband Composite Spiral Antenna for Ground Penetrating Radar

**DOI:** 10.3390/s25061890

**Published:** 2025-03-18

**Authors:** Hai Liu, Shangyang Zhang, Pei Wu, Xu Meng, Junyong Zhou, Yanliang Du

**Affiliations:** 1 School of Civil Engineering and Transportation, Guangzhou University, Guangzhou 510006, China; hliu@gzhu.edu.cn (H.L.); shangyangzhang@e.gzhu.edu.cn (S.Z.); wupei2023@e.gzhu.edu.cn (P.W.); xumeng@gzhu.edu.cn (X.M.); jyzhou@gzhu.edu.cn (J.Z.); 2 College of Civil and Transportation Engineering, Shenzhen University, Shenzhen 518061, China

**Keywords:** ground penetrating radar (GPR), spiral antenna, broadband, circular polarization, axial ratio

## Abstract

To enhance the capability of a ground penetrating radar (GPR) in subsurface target identification and improve its polarization sensitivity in detecting underground linear objects, a circularly polarized broadband composite spiral antenna was designed. This antenna integrates equiangular spiral and Archimedean spiral structures, achieving a wideband coverage of 1–5 GHz with stable circular polarization characteristics. The antenna employs an exponentially tapered microstrip balun for impedance matching and a metallic-backed cavity filled with absorbing materials to enhance its directivity. Experimental results demonstrate excellent radiation performance and stable circular polarization characteristics, with the axial ratio consistently below 3 dB throughout the operating frequency band, highlighting its suitability for polarimetric GPR systems. Furthermore, a 3D GPR measurement using the designed antenna validates its improved capacity for detecting subsurface linear objects, compared to the conventional linearly polarized bowtie antenna.

## 1. Introduction

Ground penetrating radar (GPR) is a highly effective non-destructive testing technology that has been widely applied in various fields such as civil engineering, geological exploration, and archeology [1,2]. By emitting electromagnetic waves and receiving the reflected and scattered signals from subsurface media, GPR enables rapid detection and high-resolution imaging of underground structures and targets. However, conventional commercial GPR systems generally employ a pair of linearly polarized antennas, such as bowtie or dipole types, with a fixed spacing during data acquisition [3,4]. As a result, GPR signals are only recorded in a single polarization direction. Linear objects, such as pipelines and reinforcing bars (rebars), may be undetectable in a GPR survey when their orientation is perpendicular to the antenna polarization direction [5,6]. In contrast, a circularly polarized GPR antenna possesses a greater capacity for detecting linear objects than a linearly polarized antenna.

Compared to traditional GPR, polarimetric GPR can collect richer information of subsurface targets. As a result, polarimetric GPR analyzes the relative amplitude and relative phase of reflection signals in different polarization channels for target classification. Through varying the polarization directions of both the transmitting and receiving antennas, a scattering matrix of the surface target reflection can be obtained. From the scattering matrix, various polarization attributes can be analyzed. These polarization attributes are closely related to the shape and material properties of the target and can be used to effectively differentiate underground targets, thus improving target recognition and detection accuracy [7]. Consequently, polarimetric GPR has gained increasing attention [8,9,10]. However, a field polarimetric GPR measurement using a bistatic GPR system necessitates alternately collecting data along the same survey line by changing the antenna allocation in different polarization modes [11]. To automate the data acquisition process, different types of antenna arrays have been developed in laboratories for polarimetric GPR [12,13,14]. To reduce the system’s complexity, a hybrid GPR system, which consists of a circularly polarized spiral antenna and two linearly polarized Vivaldi antennas, has been proposed [15,16]. In such a GPR system, a circularly polarized antenna with stable polarization characteristics, such as a small axial ratio (AR), over a wide frequency band is of significance for the polarimetric analysis.

Planar spiral antennas are widely employed in polarimetric GPR systems due to their excellent performance [17]. Their spiral geometry ensures consistent radiation properties and reliable circular polarization across a wide frequency range. Planar spiral antennas are typically categorized into equiangular spiral antennas [18,19,20] and Archimedean spiral antennas [21,22,23]. The arms of an equiangular spiral antenna expand exponentially with the spiral angle, resulting in excellent radiation performance and low transmission loss. But these antennas exhibit relatively inferior AR performance at low frequencies [24,25], which limits their ability to discriminate subsurface targets when they are employed in polarimetric GPR systems. In comparison, an Archimedean spiral antenna consists of arms with linearly increasing radii, possessing a compact size and superior circular polarization characteristics [26]. However, planar Archimedean spiral antennas generally suffer from a low radiation efficiency [27,28,29,30,31], which decreases signal penetration capability in GPR subsurface detection.

This paper presents a composite dual-arm spiral GPR antenna that integrates equiangular and Archimedean spiral structures to achieve broadband coverage with stable circular polarization characteristics. This antenna is equipped with an exponentially tapered balun to ensure impedance matching. Additionally, a metallic backed cavity filled with absorbing materials is employed to improve the directionality and reduce unwanted reflections from the backside. Numerical simulations and laboratory experiments are conducted to validate the improved performance of the composite antenna. The proposed antenna demonstrates significant potential for application in GPR systems.

## 2. Theory of Spiral Antenna

A spiral antenna is a frequency-independent antenna, typically composed of two symmetrically expanding arms. Its self-complementary structure maintains stable impedance characteristics with increasing arm length, resulting in wideband coverage, which is important for radar systems. Additionally, its property of stable circular polarization can improve the isolation and target identification capability of radar systems [32,33,34,35].

**Figure 1:**
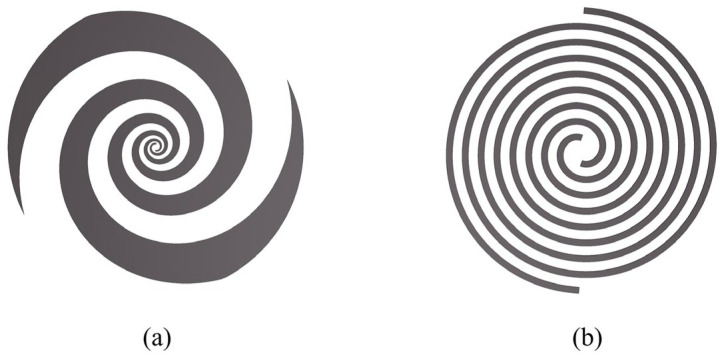
Spiral structures. (**a**) Equiangular spiral structure and (**b**) Archimedean spiral structure.

Spiral antennas are primarily categorized into equiangular spiral antennas (Figure 1a) and Archimedean spiral antennas (Figure 1b). The structure of an equiangular spiral is expressed as follows [19]:

(1) $$r=r_{0}e^{a(\theta -\theta_{0})},$$

where $r_{0}$, $\theta_{0}$, a, and θ represent the starting radius, the starting angle, the growth rate and the azimuthal angle of the spiral, respectively. At frequencies where the arm length is much shorter than the wavelength, the radiated field of an equiangular spiral antenna is linearly polarized. When the arm length approaches or exceeds one wavelength, the antenna generates circularly polarized waves. For optimal radiation characteristics and circular polarization performance, the spiral typically requires 1.25 to 1.5 turns.

The structure of an Archimedean spiral is expressed as follows:

(2) $$r=r_{0}+a(\theta -\theta_{0}),$$

where the radius increases linearly with the azimuthal angle. When the arm length exceeds one wavelength, the antenna begins to radiate efficiently. Compared to the equiangular spiral, the Archimedean spiral exhibits a tighter wound due to its linearly increasing radius, which enhances circular polarization performance and achieves wider bandwidth for a given size. However, increasing the number of turns also leads to higher transmission losses [36].

Due to the self-complementary structure of spiral antennas, according to the Babinet’s principle [37]:

(3) $${Z_{0}}^{2}=\frac{\eta^{2}}{4},$$

where *η* is the intrinsic impedance and *η* ≈ 377 Ω for air. Thus, the input impedance *Z*_0_ of a two-arm spiral antenna in free space is approximately 188.5 Ω. Typically, the presence of a dielectric substrate reduces the wave velocity along the antenna arms, resulting in a decreased input impedance [38]. However, an impedance mismatch still occurs between the antenna and the coaxial transmission line. Therefore, a balun is commonly added to achieve impedance matching.

## 3. Antenna Design

### 3.1. Composite Planar Spiral Antenna

**Figure 2:**
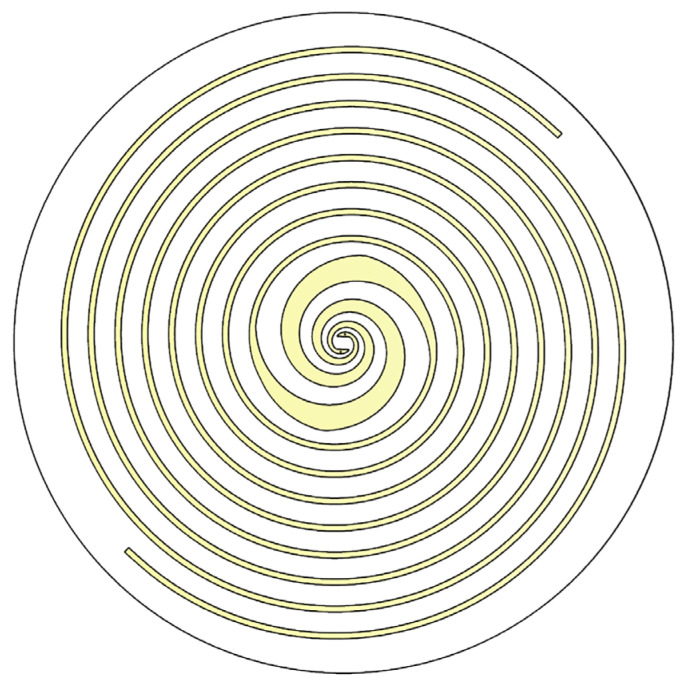
Composite planar spiral antenna structure.

The structure of the designed dual-arm spiral antenna is shown in Figure 2. This composite antenna consists of two spiral structures, i.e., an equiangular spiral in the center and an Archimedean spiral in the outer part. The equiangular spiral and the Archimedean spiral structures are connected within the same plane to form a compact dual-arm layout. The purpose of such a design is to retain the superior radiation performance of the equiangular spiral, and incorporate the good circular polarization characteristics of the Archimedean spiral in the low-frequency range, thereby improving the GPR’s capability in resolving subsurface targets.

The diameter of the designed composite spiral antenna is 110 mm. Other key parameters are given in Table 1. The composite spiral antenna was fabricated on an FR-4 dielectric substrate with a thickness of 1 mm.

### 3.2. Balun

**Figure 3:**
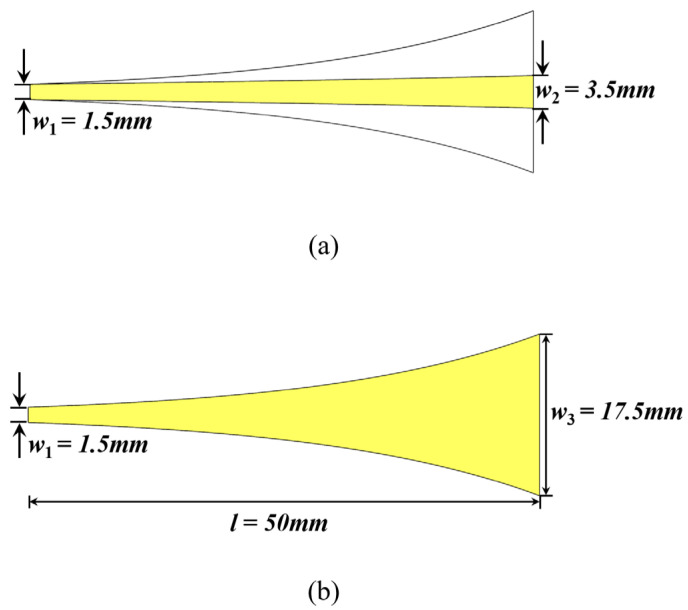
Structure of the designed balun. (**a**) Top view and (**b**) bottom view.

The designed composite planar spiral antenna features a balanced and symmetric structure with an input impedance of about 100 Ω within the working frequency band. However, coaxial transmission lines typically possess a characteristic impedance of 50 Ω. Therefore, a balun is designed for the impedance matching between the antenna and the coaxial transmission line. The balun employs a gradient line in the form of an exponential function, enabling smooth impedance transition over a wide frequency range. Figure 3 illustrates the designed balun, using a 2 mm thick FR-4 dielectric substrate. The exponential gradient microstrip line is expressed as follows [39]:

(4) $$y=\frac{w_{3}}{2}e^{\frac{\ln(\frac{w_{1}}{w_{3}})}{l}x},$$

where $w_{1}$, $w_{2}$, and $w_{3}$ represent the upper width of the balun, the lower width of the trapezoidal metal microstrip line, and the bottom width of the balun, respectively, and l is the length of the balun. Width $w_{1}$ at the balanced end and width $w_{2}$ at the unbalanced end of the balun can be determined based on the microstrip line impedance calculations as detailed in [40]. The values of these parameters are detailed in Figure 3.

### 3.3. Backed Cavity

**Figure 4:**
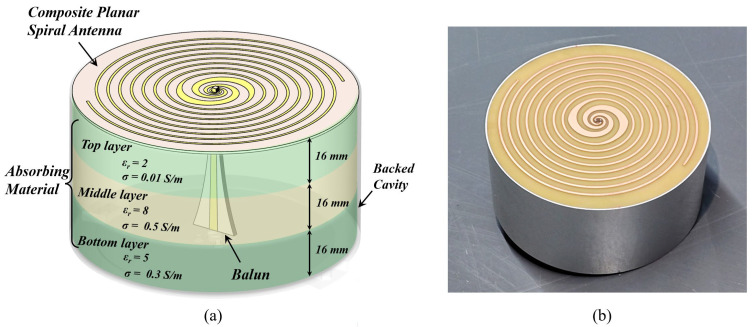
(**a**) Model of the composite dual-arm spiral antenna, and (**b**) the fabricated prototype.

Since GPR is used for subsurface detection, a metallic backed cavity structure is fixed on the rear side of the antenna to effectively suppress backward radiation. Additionally, the cavity is filled with absorbing materials to minimize multiple reflections within it, as shown in Figure 4a. The absorbing material consists of three layers with distinct dielectric properties. The top layer employs materials with a low relative permittivity and conductivity. It reduces the reflection of electromagnetic waves at the interface, allowing greater penetration of electromagnetic waves into the absorbing material. The middle layer utilizes materials with high relative permittivity and conductivity, enhancing the attenuation and absorption of electromagnetic waves within the material. The bottom layer incorporates materials with moderate relative permittivity and conductivity to balance reflection and absorption, thereby reducing mismatched reflections at the interface within the metallic cavity [41].

## 4. Antenna Evaluation

Electromagnetic simulations of the designed composite planar spiral antenna were conducted to evaluate its performance. The simulation model for the absorbing material was established based on reference [41], as shown in Figure 4a. In addition, a prototype of the antenna was fabricated for testing, as illustrated in Figure 4b. Then, the return loss and transmission performance of the fabricated antenna were tested using a Vector Network Analyzer (VNA) in an open area to minimize environmental reflections and ensure accurate characterization of the antenna’s radiation properties.

**Figure 5:**
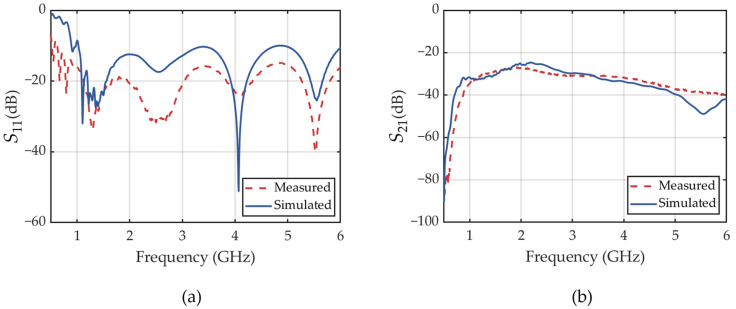
Measured and simulated *S*_11_ (**a**) and *S*_21_ (**b**) of the antenna (in dB).

Return losses (*S*_11_) is a critical parameter for evaluating antenna impedance matching. It is defined as the ratio of the reflected energy to the incident energy at the antenna port and is generally expressed in dB. A lower *S*_11_ value indicates better impedance matching, and a threshold of *S*_11_ < −10 dB is widely accepted as indicative of good matching characteristics [42]. The measured and simulated *S*_11_ of the designed antenna in free space are shown in Figure 5a. The measured results agree well with the simulations, indicating an effective bandwidth ranging from 1 to 6 GHz. This broad bandwidth enhances GPR imaging resolution for subsurface target characterization. In comparison, the measured *S*_11_ is lower than the simulated result, which can be primarily attributed to differences in the dielectric properties of the absorbing material used in the numerical model and the real antenna. Both the measured and simulated results exhibit excellent matching characteristics, confirming the reliability of the antenna design.

**Figure 6:**
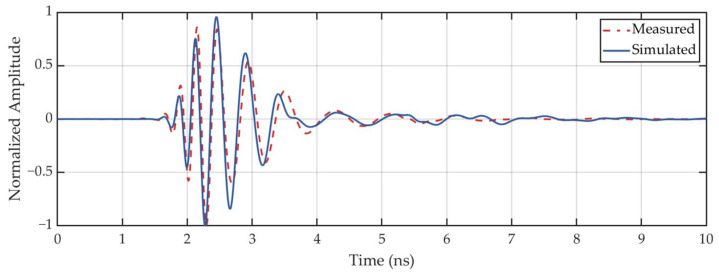
Normalized time-domain waveforms (*S*_21_).

Then, we conducted a transmission measurement (*S*_21_) and the corresponding simulation by placing two left-hand circularly polarized (LHCP) antennas 0.5 m apart along the boresight axis of each other. The obtained frequency spectra and time-domain waveforms of the transmission signals are illustrated in Figure 5b and Figure 6, respectively. The measured transmission spectrum agrees well with the simulated result. A stable transmission performance of the antenna over the frequency range from 1 to 5 GHz is verified. It is also evident that the measured and simulated time-domain waveforms exhibit good agreement, with only minor discrepancies in amplitude. Additionally, because the spiral antenna is a typical dispersive antenna [36], the high-frequency components of the transmitted signal arrive prior to the low-frequency components, degrading the imaging resolution.

**Figure 7:**
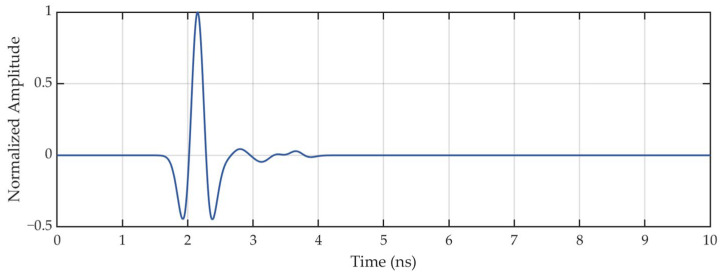
Time-domain waveform after deconvolution processing.

To mitigate the impact of the antenna dispersion, we apply a deconvolution technique to the transmitted waveform. Specifically, we utilize a matching pursuit algorithm [43] to perform deconvolution. The details of the algorithm are explained in Appendix A. This method deconvolves the received signal by iteratively identifying and removing the correlated wavelet components to recover the reflection coefficient sequence and then convolving the recovered sequence with a template wavelet. Figure 7 illustrates the deconvoluted waveform, showing a significant reduction in dispersion-induced distortions.

**Figure 8:**
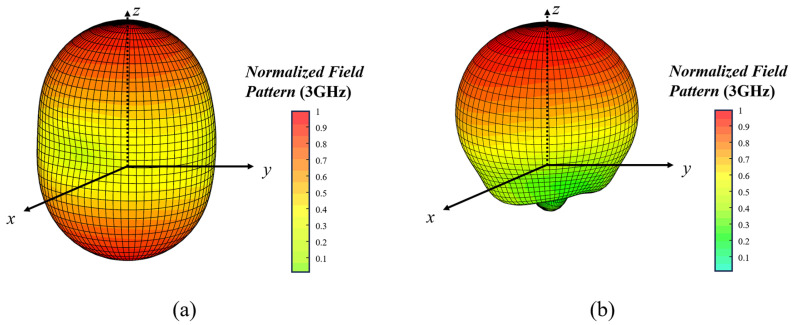
Normalized three-dimensional radiation pattern of antenna. (**a**) Without backed cavity and (**b**) with backed cavity.

We further evaluated the effectiveness of the backed cavity through simulations, and the resulting radiation patterns at an operating frequency of 3 GHz with and without the cavity are shown in Figure 8. It can be observed that the radiation pattern of the antenna without the backed cavity is bidirectional, and axially symmetric along the normal direction of the spiral plane. In contrast, the absorbing material inside the backed cavity absorbs most of the energy radiated backward, ensuring that the antenna exhibits good directivity.

Axial ratio (AR) is an important parameter for evaluating the polarization characteristics of an antenna, which is defined as the ratio of the electric field strengths in the orthogonal major and minor axes of the polarization ellipse when the antenna transmits or receives elliptically polarized electromagnetic waves. The definition of the axial ratio is described as follows [36]:

(5) $$AR=20\;\log_{10}(\frac{E_{major}}{E_{\mathrm{minor}}}),$$

where *E*_major_ and *E*_minor_ represent the electric field strengths in the major and minor axes directions of the electromagnetic wave, respectively. When the AR is 0 dB, it indicates a perfect circular polarization state. The smaller the AR, the better the antenna’s circular polarization performance. In polarimetric GPR systems, superior circular polarization can improve their capability for subsurface target discrimination. To achieve a good circular polarization performance, the AR is typically required to be below 3 dB across the working frequency band [44].

**Figure 9:**
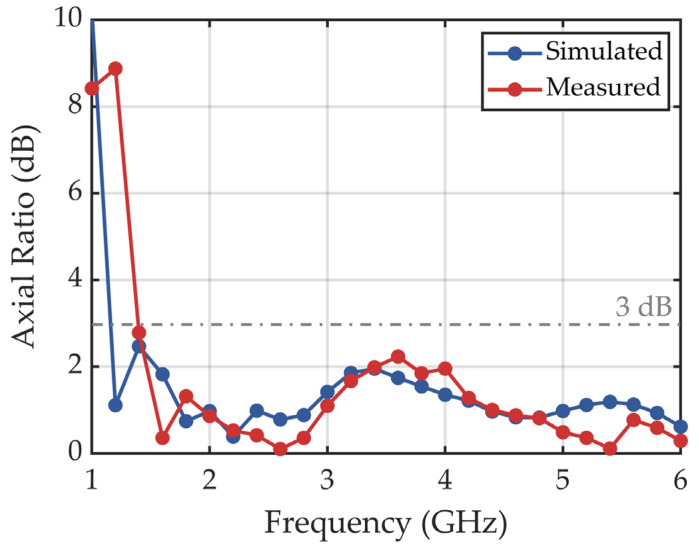
Measured and simulated AR of the designed composite spiral antenna.

To test the AR of our designed antenna, a linearly polarized Vivaldi antenna was used as the receiver, and the designed antenna was used as the transmitter in the measurement. The transmitting and receiving antennas were placed 0.5 m apart along their respective boresight axes. The receiving antenna was rotated 360° during the test, and the electric field strength *E* was recorded at each frequency with an interval of 1.8° [45]. The simulated and measured results of AR are shown in Figure 9, demonstrating a good agreement. The AR of the designed antenna remains below 3 dB across the 1–6 GHz frequency band, indicating excellent circular polarization performance.

Table 2 compares key performance metrics of different spiral antennas. The proposed antenna demonstrates the highest fractional bandwidth of 142.9%, and the best circular polarization performance (axial ratio < 3 dB) across 1–6 GHz. Its compact size and wide frequency coverage make it highly suitable for GPR systems.

## 5. Three-Dimensional GPR Measurement

**Figure 10:**
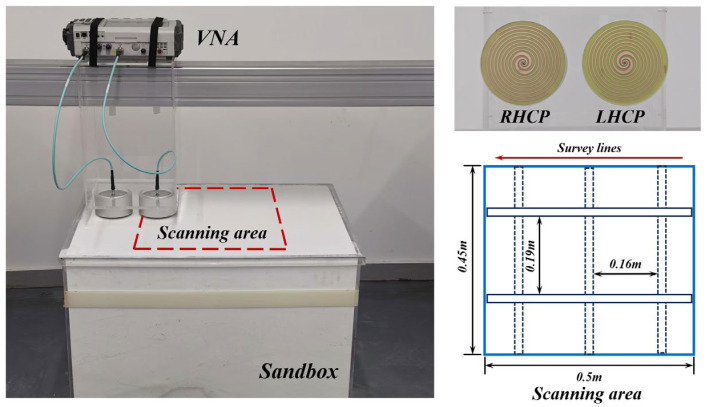
Three-dimensional GPR measurement using the fabricated spiral antenna.

**Figure 11:**
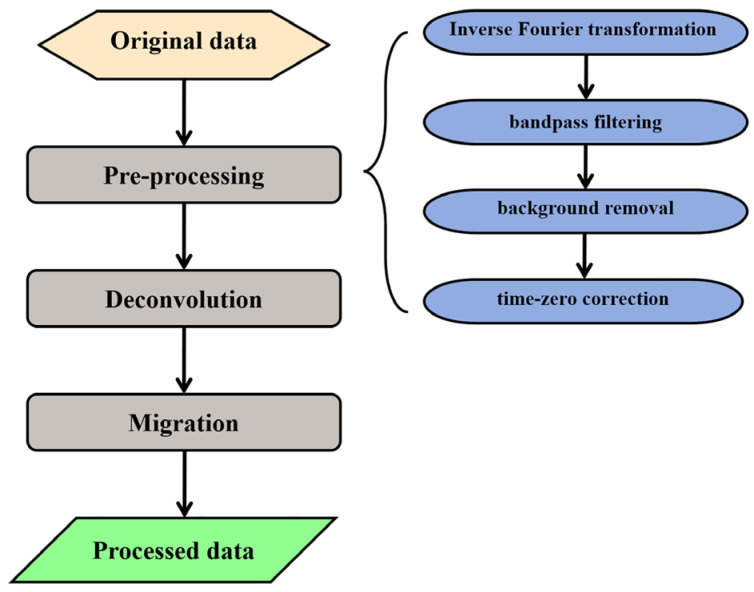
Flowchart of the data processing.

To evaluate the performance of the designed antennas for detecting subsurface linear objects, we established a GPR system comprising a VNA, a LHCP antenna, and a Right-Hand Circularly Polarized (RHCP) antenna, as shown in Figure 10. Five rebars with a diameter of 1 cm were buried in a sandbox at a depth of 12 cm, where the sand has a relative permittivity of 3. The scanning area of the 3D GPR measurement was 0.45 × 0.5 m^2^. GPR signals were recorded by the VNA over the frequency range from 5 MHz to 6 GHz with a sampling number of 1200, and a total of 31 survey lines were measured, with an equal line spacing of 0.015 m. The recorded GPR data are pre-processed, including inverse Fourier transformation, bandpass filtering, background removal, and time-zero correction [2]. Additionally, deconvolution processing is performed to compress the wavelet for resolution enhancement. The data processing flowchart is illustrated in Figure 11.

For comparison, we also collected 3D GPR data using a commercial dual-polarization GPR system, IDS C-Thrue with a center frequency of 2 GHz. This system is equipped with two pairs of linearly polarized antennas in the horizontal and vertical directions, allowing for simultaneous data collection in both the HH and VV polarization channels. The data processing workflow is identical to that used for the data recorded by our fabricated GPR system.

**Figure 12:**
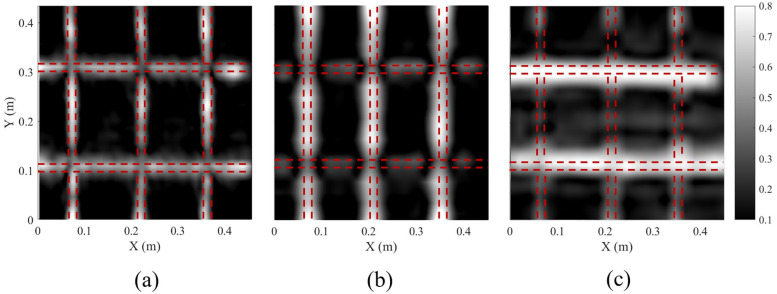
Depth slices of the rebars reconstructed by (**a**) the configurated GPR system in Figure 9, and (**b**) the HH and (**c**) VV polarization channels of the commercial GPR system. Dashed lines indicate the positions of the five buried rebars.

Figure 12 shows the reconstructed depth slices of the rebars after migration [46] from the 3D GPR data recorded by both the fabricated spiral antennas and the commercial GPR system. It can be observed that in the HH channel of the commercial GPR system, reflections from the two horizontal rebars in the upper layer are clearly visible, while reflections from the vertical rebars in the lower layer are weak. Similarly, in the VV channel, the three vertical rebars are distinct, while the two horizontal rebars are hardly distinguishable. It indicates that the linearly polarized bowtie antenna used in the commercial GPR system is only sensitive to the rebar oriented in the polarization direction of the antenna. As such, two GPR surveys in orthogonal directions are usually conducted to detect rebar net [47], making the field measurement time-consuming. In contrast, all five rebars are clearly imaged in the depth slice using the manufactured spiral antenna. The amplitudes of the rebar reflection in two directions are almost the same. These results further validate the good circular polarization performance of the spiral antenna. Additionally, due to the broader bandwidth, the imaging resolution of rebars using the developed spiral antenna is significantly superior to that achieved by the commercial GPR system.

## 6. Conclusions

This paper presents a composite dual-arm spiral antenna for GPR. The designed antenna achieves a wideband coverage from 1 to 6 GHz while maintaining stable circular polarization performance, with the AR remaining below 3 dB across the operating frequency band. The incorporation of a metallic backed cavity with absorbing materials enhances its directivity and minimizes unwanted GPR reflections from objects above ground. Furthermore, laboratory experiments have demonstrated the antenna’s capability to improve detection accuracy and reliability in real-world GPR applications. Future work will focus on applying the composite spiral antenna to developing polarimetric GPR systems to improve their capability for the detection and characterization of subsurface targets.

## Figures and Tables

**Table 1:** Parameters of the designed composite spiral antenna.

	Equiangular Spiral Structure	Archimedean Spiral Structure
Starting radius (*r*_0_)	1.5 mm	14 mm
Starting angle (*θ*_0_)	0°	0°
Azimuthal angle (*θ*)	720°	1800°
Growth rate (*a*)	0.221	9
Line width	-	1 mm

**Table 2:** Comparison of different spiral antennas.

Reference	*S*_11_ Bandwidth (GHz)	AR	Size (mm^2^)	Fractional Bandwidth (%)
[20]	3.95~10.6	>3 dB in the whole band	59.94 × 59.94 × 1.14	91.4
[23]	0.8~3.5	<3 dB (0.5~2.5 GHz)	152.4 × 152.4 × 15	125.6
[26]	2.5~4.5	<3 dB (2.5–4.5 GHz)	38.2 × 38.2 × 10	57.1
[28]	1.9~8.5	<3 dB (2–6 GHz)	36 × 36 × 20	126.9
[31]	0.8~3	<3 dB (1.5~3 GHz)	76.2 × 76.2 × 38.1	115.8
[39]	5~20	<5 dB (5~20 GHz)	50 × 50 × 23	120
This work	1~6	<3 dB (1~6 GHz)	111.5 × 111.5 × 51.8	142.9

## Data Availability

Data are contained within the article.

## Abbreviations

The following abbreviations are used in this manuscript:

GPR	Ground Penetrating Radar
VNA	Vector Network Analyzer
LHCP	Left-Hand Circularly Polarized
RHCP	Right-Hand Circularly Polarized
AR	Axial Ratio

## Appendix A. Deconvolution Method

The received signal ***y*** can be regarded as the convolution of the reflection coefficient ***x*** and the wavelet ***h***.

(A1) y=h∗x,

where ∗ denotes the convolution operation. The original wavelet ***h***_s_ can be obtained by extracting a complete echo from ***y***, with the length of ***h***_s_ is m. The dictionary matrix ***D*** can be constructed as follows:

(A2) $$D_{i,j}=\left[\begin{array}{cccc} h_{s(1)} & 0 & \cdots & 0\\ h_{s(2)} & h_{s(1)} & \cdots & 0\\ \vdots & \vdots & \ddots & \vdots \\ h_{s(m)} & h_{s(m-1)} & \cdots & 0\\ 0 & h_{s(m)} & \cdots & 0\\ \vdots & \vdots & \ddots & \vdots \\ 0 & 0 & \cdots & h_{s(m)} \end{array}\right],$$

where ***D*** represents all possible time-shifted versions of the wavelet ***h***_s_, and each column is called an atom. Here, *i* denotes the length of ***y***. This construction enables sparse representation of the signal through linear combinations of these atoms.

We initialize the sparse coefficient ***x***^(0)^ as a zero column vector of length *j*. At each iteration *k*, the algorithm selects the atom from the dictionary ***D*** that exhibits the maximum correlation with the current residual vector ***r***^(*k*−1)^. The residual vector ***r***^(*k*−1)^ represents the difference between ***y*** and the current sparse approximation ***Dx***^(*k*−1)^. Specifically, the index *i_k_* of the selected atom is determined by the following:

(A3) $$j_{k}=\underset{j}{\arg\max}\left|D_{:,j}^{⊤}r^{\left(k-1\right)}\right|,$$

where ***D****_:,j_* represents the *j*-th atom in the dictionary and ⊤ denotes the transpose operation.

The sparse coefficient ***x***^(*k*)^ at the *j_k_*-th position is updated as follows:

(A4) $$x^{\left(k\right)}[j_{k}]=D_{:,j_{k}}^{⊤}r^{\left(k-1\right)},$$

and the residual is updated by subtracting the contribution of the selected atom as follows:

(A5) $$r^{\left(k\right)}=r^{\left(k-1\right)}-D_{:,j_{k}}x^{\left(k\right)}[j_{k}],$$

When the *l*_2_-norm of the residual satisfies the following:

(A6) $${\left\|r^{\left(k\right)}\right\|}_{2}<\epsilon ,$$

where *ϵ* represents the residual threshold, the iteration stops. Here, *ϵ* is typically set as a small fraction of received signal energy or derived from noise statistics.

The reflection coefficient ***x*** is then reconstructed as follows:

(A7) $$x_{\mathrm{rec}}=\sum_{t=1}^{k}x^{\left(t\right)}[j_{t}]e_{jt},$$

where ***e****_jt_* represents the standard basis vector. Then, the reconstructed reflection coefficients ***x***_rec_ are convolved with the template wavelet ***h***_t_, the reconstructed signal can be obtained as follows:

(A8) $$y_{\mathrm{rec}}=h_{t}*x_{\mathrm{rec}}$$
